# The expanding role of robotics in the clinical laboratory

**DOI:** 10.1155/S1463924692000038

**Published:** 1992

**Authors:** K. Ozawa, P. Schnipeslky, H. L. Pardue, J. Place, A. Truchaud

**Affiliations:** 1 Bio-Medical Center Naka Works Hitachi Ltd, 882 Ichige Katsuta 312 Japan; 2 PH.D. Bio-Medical Center Naka Works Hitachi Ltd 882 Ichige Katsuta 312 Japan

## Abstract

This paper provides an in-depth description of the current applications of robotics in clinical laboratories. The trends and impact of the use of robotics in clinical chemistry in the forseeable future are also discussed.


Journal of Automatic Chemistry, Vol. 14, No. 1 (January–February 1992), pp. 9–15

### The expanding role of robotics in the clinical laboratory

**K. Ozawa, P. Schnipeslky, H. L. Pardue, J. Place and A. Truchaud for the International Federation of Clinical Chemistry**
*Bio-Medical Center, Naka Works, Hitachi Ltd, 882, Ichige, Katsuta, 312 Japan*

*This paper provides an in-depth description of the current applications of robotics in clinical laboratories. The trends and impact of the use of robotics in clinical chemistry in the forseeable future are also discussed.*

### Introduction

The world of science fiction abounds with intelligent robots possessing fantastic abilities. They can move about—some can even fly. They have skilful hands and powerful arms. They can communicate with each other and can also converse with human beings. They think like humans.

In reality, the mechanical functions of robots resemble those of people, but they usually have little or no intellectual capacity. They repeat preprogrammed tasks, although they can be reprogrammed if necessary. Generally, they have only one arm, equipped with two fingers or grips, and they cannot usually move around in their working places. Most of them are firmly fixed on rigid bases and can only access a limited area. The application of robots is often limited by the degree of flexibility of their hands, and a variety of replaceable hands may be needed for each different task.

In addition to the fixed type of robot, some mobile systems have been developed. These are either used for educational or novelty purposes, such as robotic tourist guides in amusement centres, or for more serious tasks like carrying heavy or dangerous materials in factories.

This paper describes the present applications of robotics in clinical laboratories, and discusses the trends and impact of the use of robotics in clinical laboratories in the not too distant future.

### Definition

As described above, a variety of systems exist and robots can also be defined in a number of ways. One definition, developed by the Robotics Industries Association, states: 'An industrial robot is a reprogrammable multi-functional manipulator designed to move materials, parts, tools, or specialized devices through variable programmed motions for the performance of a variety of tasks'.

Instead of looking at automated systems from this mechanical point of view, they can be considered more as computer-based systems, equipped with sensors and motors or actuators. In its simplest form, the actuator is connected to the sensor and the sensor can receive feedback that may initiate a new cycle of action. The system is then a machine capable of repeated action – an 'automaton'. The earliest automatons, for example clocks, were entirely mechanical. With the introduction of programmable software it became possible to introduce a large degree of flexibility and develop the automated systems we know today. When the system is so flexible that it is capable of the manipulations described in the Robotics Industries Association definition, we can call it a robot.

The essential elements of the robot that appear to differentiate it from the automaton are its flexibilty and its capability for humanlike manipulation. However, there is no clear distinction between the two.

Another potential advantage of the introduction of programmable software is the opportunity to introduce intelligence into the system. Like a human, an intelligent robot will not only work according to given programs but will correct its movement or work with reference to its environment. The robot will remain unintelligent so long as it is not equipped to do this. The intelligent robot therefore needs to be equipped with additional sensors and feedback capabilites, or recognition systems, that allow it to appreciate its situation or environment. Then the addition of decision-making capability in the form of expert systems adds the possiblity of recovery, interaction with the human operator and process optimization.

On the other hand, standard flexible manufacturing or machining systems are almost completely unaware of their environment, they cannot make a diagnosis of the results of what they have done, or solve problems. They cannot change or correct their operations properly. Intelligent robots are able to go beyond these problems and cope with complex situations.

### Configuration

Because industrial robotics, as a discipline, centre on the automation of mechanics, and is concerned with movement functions, manipulators are often classified into three categories according to their mechanical configuration or co-ordinate system (figure 1). Three categories

Comments on this paper and requests for offprints should be sent to: Kyoichi Ozawa, PH.D. Bio-Medical Center, Naka Works, Hitachi Ltd, 882 Ichige, Katsuta, 312 Japan

© IFCC 1992

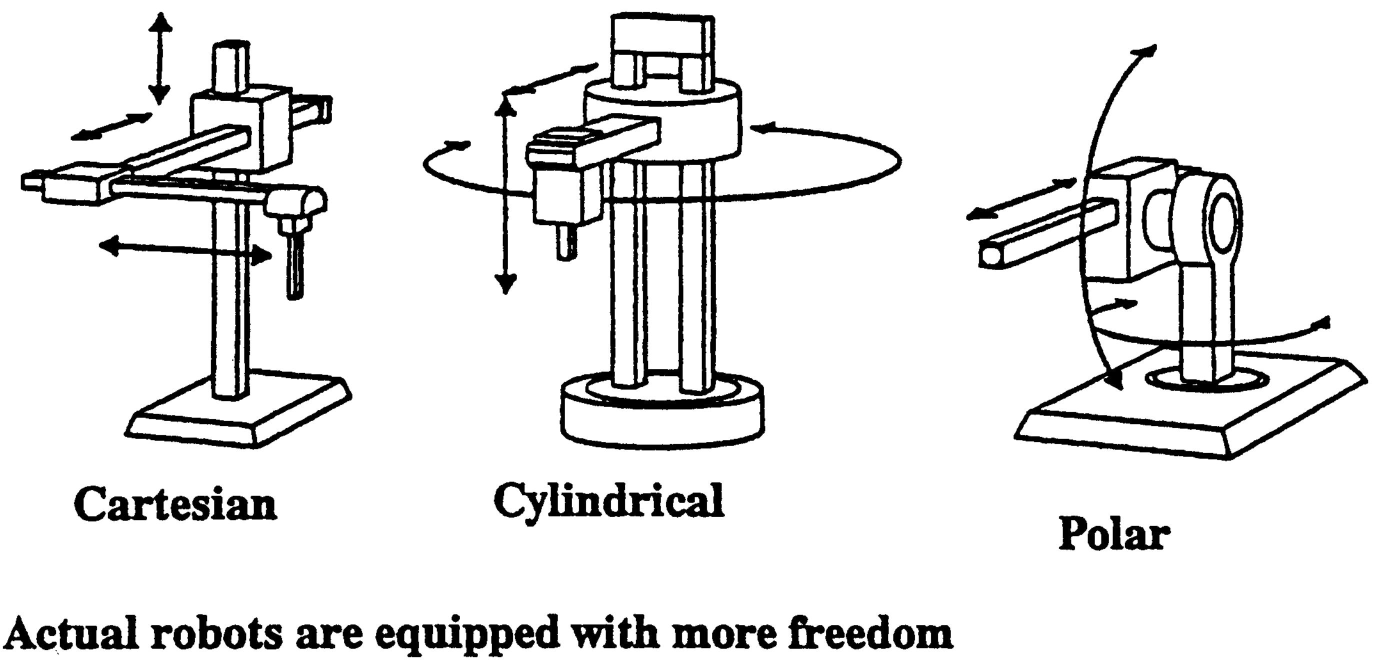

*Figure 1. Typical co-ordinate system of robotics.*

commonly encountered are Cartesian (or x-y-z), cylindrical and polar. Extra degrees of freedom in a spherical or revolving configuration are obtained by adding extra joints to the arm. More recently such manipulators have been equipped with tracks on which they can move backwards and forwards to extend their access area [1]. This puts more emphasis on the avoidance of collisions and requires extra tactile or proximity sensors.

Most manipulators have a wrist assembly at least capable of rotation, pitch, and yaw. In its most primitive form, the gripper assembly consists of a pair of fingers that close to grasp an object, but more developed forms require sensor feedback to allow the manipulator to perform many of the human arm and finger-pair operations.

The greater number of degrees of freedom and the requirement for extra sensors and feedback increases the need for intelligent control. It is as this intelligent control is added to the manipulator that it finally becomes well differentiated from the automaton. Intelligent robots are thus characterized by the fact that they can sense and recognize objects in their access area. This might be accomplished, for example, by using a video camera coupled to a shape recognition system.

### Current situation

Automation is particularly useful in hazardous environments, in situations where a human operator cannot or should not be present. Large numbers of robots are applied effectively in working environments that are especially dirty, noisy or hazardous. For example in automobile factories the working scene of welding robots emitting sparks, or of painting robots spraying, or of assembly robots working accurately at quite high speed is very familiar. Robots are also being developed for particularly dangerous environments, such as spray-coating of fire-preventive material in buildings, removing rust from the bottom of ships, and for work in active areas of nuclear reactors.

Examples in the clinical laboratory are to be found in the manipulation of reagents containing isotopes or carcinogens and in the handling of potentially biohazardous samples, for example those which might contain infectious viruses.

Another application area for robotics is for tasks requiring high precision and cleanliness together with repetitivity. In semi-conductor manufacture, and in the assembly of printed circuit boards, robotics can replace human operators for whom repeated and precise operations can be exhausting. In the clinical laboratory an example of this kind would be the injection of large series of sample in a chromatograph.

Automatons dedicated to given purposes are most suitable for mass production of a few models over a long period of time and can be more productive and economical than robotic systems. The equivalent of automatons in clinical chemistry are automatic analysers, and these can effectively deal with many steps in the analytical processing of specimens that inundate the laboratory.

Laboratory automation is widely used in industry. Most industrial chemical laboratories carry out a range of routine chemical determinations on a large number of samples. Once the analytical methods are fully established, their execution is a boring and monotonous task and dedicated analysers are used.

Why are robots used for automation instead of standard automated machining or manipulating systems? The key issue here is the flexibility of robots. Robots can be reprogrammed after the product design is changed, and even after the product is remodelled and the assembly line rearranged completely. If the project or the chemical process involved is going to be modified or changed frequently, a robot is eminently suitable, whereas a dedicated system is not.

As a result , there will continue to be a place for robots in the dedicated automated production line. Similarly, robots are well suited to development projects with rapidly changing parameters.

The diagram shown in figure 2, which was orginally

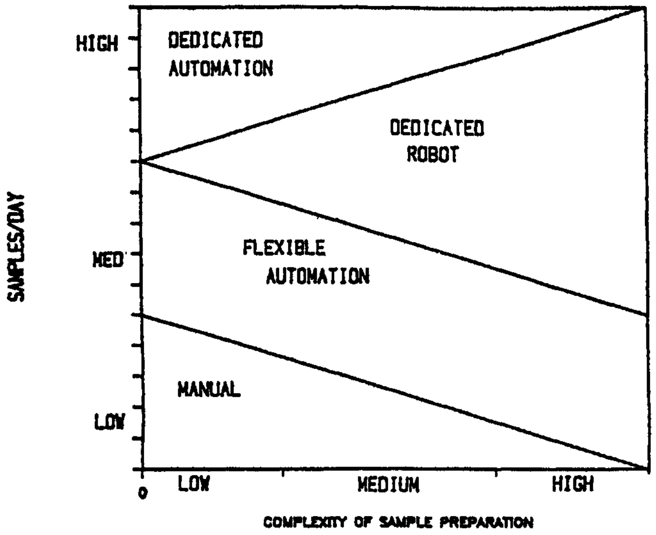

*Figure 2. Automation and robotics system (after Felder).*

proposed by Zenie [2] and modified slightly by Felder [3] illustrates where robotics or dedicated automation would be the appropriate choice. The illustration, with its two axes, is appropriate now, but might be subject to changes depending on the economics of robotics systems and their availability.

### Communication and language

### *Language*

To employ the robotics system in laboratories it is important to have an easy language, which is not so different from the natural language, and can express complex series of tasks common of laboratory in simple way.

The most successful and typical applications of robotics are found in batch mode tasks. The tasks consist of rather simple components of easy, monotonous and repetitive processes. No really intelligent robots are available at this time. For example, there is no robot on the market which manages complex work by commands written in a natural language, such as 'Give me the LDH activity of each of 34 serum samples in the test tubes in the racks A on the table B by 2 p.m.'. Today's robots need much more precise and detailed descriptions of the process that should be completed. The processes have to be broken down into single movements, such as 'move arm A from position B (Xb, Yb and Zb) to position C (Xc, Yc and Zc)', or 'rotate arm D a certain (E) degree clockwise from F degree'.

A considerable effort has been spent on creating a high-level language which is easily understandable and can be written by laboratory technicians who are not familiar with so-called 'low-level' languages. Some progress has been made. However, a standardized language [4], standard protocols for communication and standard interface hardware between robots, instruments and a computer or a sequencer [5], similar to the 'MIDI' system for musical instruments [6], have to be established as soon as possible, before many robotic systems of various types, concepts and protocols are used. This would allow many types of robots with languages that are not presently compatible with each other to be coupled together and employed widely in laboratories. MIDI is an abbreviation of Musical Instruments Digital Interface and was developed as a standard for hardware interfaces and communication protocols between electronic musical instruments, sequencers, effectors, mixers and other components. MIDI is being used worldwide by musical instrument makers, sequencer makers and also by computer hardware manufacturers and software shops. Using MIDI, musical instruments and other components can communicate with each other through DIN cables and work together.

The programming of a smart robotic system will be difficult, but once the robot is programmed properly for a given task, the operation of the robot itself is another story and is usually not so difficult for a technician untrained in robotics.

### *Workcell concept*

Kramer proposed a 'workcell' concept [7]. The simple workcell is defined as an intelligent component that allows samples to be transported in and out, allows data to be up- or down-loaded, and allows external control from and provides internal status to outside devices. Complex workcells will work as a group of several simple workcells of the same or of different kinds to do a specific task or series of tasks.

Kramer also predicts that in the next ten years we will see the development of instrument-friendly network interfaces for analytical equipment, replacing the efforts of the past 10 years to create human user-friendly interfaces for analytical equipment. In other words, we will also see the development of 'robotic-friendly' interfaces for analytical equipment.

### *Standardization of interfaces and communication protocols*

Proposals need to be made for the standardization of interfaces and protocols for communication between devices – robots, instruments and their control modules. This standardization will allow any one device to be connected to another, or integrated into a single system, regardless of its model and its make, and will lead us to

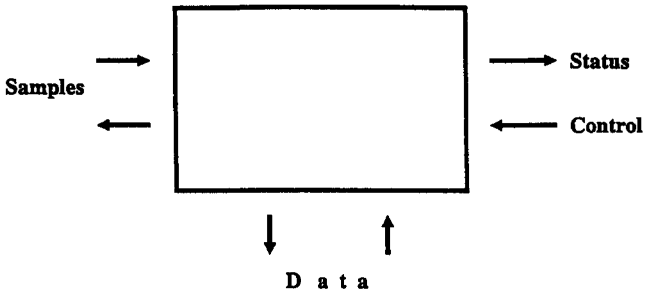

*Figure 3. Kramer's workcell concept.*

develop a universal and high-level, standard language that is easily written and understood by people with no special expertise in this field.

These proposals need to include and define the following items.

*Physical requirements*

Physical requirements are the requirements needed for designing hardware.

(1) *Transmission media – electrical, optical:* Signals, commands and information to and from devices will be transmitted as electrical or optical signals.

(2) *Transmission line – wire, optical fibre:* The electrical signals will be transmitted through electrical wire and the optical through optical fibre.

(3) *Network topology – star, ring/loop, bus:* If we have many devices to make a system, there are several ways to connect those devices each other.

Star: Usually a master controller will be placed at the centre of an imaginary star with many radial connections, with each slave device placed at the periphery and connected to the master directly.

Ring/loop: These two words are sometimes used to mean the same thing. In this system each device will be connected to a ring or a loop-shaped way and can transmit or receive its signal in successive order.

Bus: Several signals of different origins from one device are sent through individual lines. So there will be a bundle of parallel lines of signals. This kind of signal path is called a bus, or highway, or trunk.

(4) *Transmission – serial, parallel:* Serial transmission of a signal can occur through one or several lines of signal. For the bus we need or are able to do parallel transmission of signals.

(5) *Modulation:* There are many ways to transmit signals, as there are in telecommunications. Usually signals will be transmitted as coded pulses.

(6) *Signal level:* For electrical signals, current, and/or voltage, level must be defined. For optical signals the wavelength, source and intensity of the light have to be defined.

(7) *Junction hardware – connector:* There are many connectors on the market and suitable connectors need to be selected for each type of connection application. Pin assignments also need to be made for each proposed connector.

*Logical requirements*

Logical requirements are the requirements needed for coding and for the communication protocol between devices.

(1) *Coding:* There are several ways of coding, but the most common are:

Binary: Each number being expressed in powers of two. The most basic and efficient coding but hard to read.

ASCII: American Standard Code for Information Interchange.

EBCDIC: Extended Binary Coded Decimal Interchange Code.

(2) Basic transmission protocol: *Handshake* – this means that one device asks permission of the other for transmission of signals. When an OK is received, it will make the transmission. The protocol of the handshake has to be defined.

(3) *Command for robot's movements:* Any command to be understood by devices should be readable and easily understandable by humans.

### Applications in the clinical laboratory

*History*

In chemical laboratories, increasing numbers of robots are being employed for sample preparation, to reduce monotonous tasks or to avoid human exposure to dangerous environments. Robots used in conjunction with computer systems can provide more reproducible and reliable results. In clinical chemistry, this situation is slightly different; there are few examples of robotics employed in the clinical laboratory.

In industrial chemical laboratories, robotic systems are able to carry out a number of experiments under slightly differing conditions to find the most suitable conditions, for example for organic syntheses or for picking up a colony of micro-organisms which produce the most effective target antibiotics from a large number of soil samples [8]. This laboratory work cannot be done effectively by technicians, because it is tedious. And often the number of experiments will be far beyond the capacity of the normal laboratory.

Clinical chemical laboratories are rather different. Although there is an apparent trend for a continuing increase in the number of samples and in the number of different analytical procedures, most determinations and procedures are kept uniform for a long period of time. One of the key reasons for keeping procedures unchanged is to avoid confusing doctors by making changes in

*Table 1. Robotic application fields and unit operations.*

| | Robotic application | | | | | | | | | | |
|---|---|---|---|---|---|---|---|---|---|---|---|
| Unit operation | (1) | (2) | (3) | (4) | (5) | (6) | (7) | (8) | (9) | (10) | (11) |
| Weighing or pipetting | X | X | X | X | X | X | X | X | X | X | X |
| Internal standard addition | X | X | X | | | | | | | | |
| Dilution | X | X | X | X | X | | X | | | | X |
| Reagent addition | X | X | X | | | X | | | | | X |
| Mixing | X | X | X | X | | | | | | | |
| Injection to sample port | X | X | | | | | | | X | | |
| Centrifuge | X | X | X | | | | | | | | |
| Filtration | X | X | X | | | | | | | | |
| Sip to photometer | | | | X | | | X | | | | |
| Washing | | | | | | | X | | X | | |
| Antigen coating | | | | | | | | | | X | |
| Transportation | X | X | X | | | | | | | X | |
| Capping and uncapping | X | X | X | | | | | | | | X |
| Heating | | | | | | X | | | | | |
| Extraction | | X | X | X | | | | | | | |

(1) HPLC; (2) GC; (3) TLC; (4) UV/VIS; (5) pH; (6) Hydrolysis; (7) Dissociation; (8) Water content; (9) Viscosity; (10) ELISA; (11) Inoculation.

analytical procedures that might require a re-evaluation of medical significance.

For biochemical analysis of blood, fully dedicated automated chemical analysers of various types and sizes are extremely effective in terms of keeping the cost of analysis constant. This is the main reason for there being little demand for robotics in the clinical chemical laboratory.

Table 1 shows some examples of application fields for robotics and the operations they perform. These are rather simple examples and there must be many other jobs that are well suited to the employment of robotics.

*Introduction of robotics in the clinical laboratory*

Robots could be introduced to clinical laboratories in many ways. For example, for the preparation of specimens and sorting to various destinations, for placing samples or specimens in and removing them from a centrifuge, for transportation, for aliquotting to analysers, sorting for storage and to handle wastes.

Robotics could be used in clinical laboratories to optimize workflow and increase throughput, to shorten turnaround time, and to manage with fewer full-time staff equivalents [9], or to reduce chances of exposure to possible biohazards.

Robotics were first applied to clinical laboratories in the 1980s. In 1981 Seligson of Yale-New Haven Medical Center reported the introduction of a robot carrier for transporting clinical specimens [10]. The robot moved 300 ft to its furthest destination in 2.5 min, and operated 24 hours a day. Batteries were changed twice a day by laboratory personnel. It used a sonar sensor to look for people or obstacles in its path, slowed and then waited for them to move. This was apparently the first presentation on robots to appear in *Clinical Chemistry.*

The second presentation on robots in *Clinical Chemistry* was presented by Hawk in 1983 [11]. The system is arranged for liquid handling, sample conditioning and separation. Thus it is mostly used in sample preparation or for 'front end automation'.

In the Kochi Medical School, Sasaki employed cylindrical working robots in analytical procedures for blood transfusion and hormone assay [12,13,14,15]. They do pipetting, dilution, stirring, transportation of various aliquots etc. Several supporting parts, or devices, such as micro-titre-plate supplier, pipette tip supplier and reagent reservoirs, are placed around the robots within their accessible range. This provides for speedy and accurate analysis and also protects technicians from potential biohazards. The Kochi robots are operated using a 16 bit personal computer and programmed with a language similar to BASIC.

In addition, several robotic-like manipulators are being used in the 'belt-line system' built in the Kochi Medical School [16]. These manipulators connect almost all analytical instruments and devices in the laboratory to the belt-line system and allow the transfer of all specimens to every destination. They are not commercially available robots, but specially designed manipulators and connecting devices between the belt-line and the automated analytical equipment. For example, one of these robotic mechanisms picks up one urine test strip out of a specially designed strip container, dips the strip into urine in a specimen container and then places the strip on the tray of an automatic urine analyser. The analyser will do the rest of the procedure for urine analysis and then report or send the analytical results to the computer system that supervises the whole system and collects the results. This series of movements is triggered by a signal issued when each urine specimen container arrives at the urine analysis position on the belt line system.

The Kochi system is probably the most sophisticated example of application of robotics in the clinical laboratory.

Felder and his group [3,17,18,19,20,21,22] have constructed an unmanned, robot operated laboratory equipped with a multi-jointed robot (polar co-ordinate), a blood gas analyser, a blood electrolyte analyser and a touch-screen computer terminal. Several systems are placed in close proximity to critical patient care areas and monitored by a central computer through a local area network. From a biosafety standpoint, this robotic system is very helpful because it handles a specimen container which is usually a syringe with a sharp needle – a risk when handled manually, even with gloved hands. This is a clever example of a system which is the result of the combined work of several robots and a single computer supervisor.

### Discussion

Robotics will be employed in various ways in many parts of the clinical laboratory and their numbers will steadily increase.

Extensive planning is needed to make the employment of robots successful. Thorough examination and analysis of procedures being automated will be important to start the planning process. Cross has described criteria which help to make robot applications successful [23]. He analysed the process of tasks and emphasized the necessity of endless patience to debug and customize programs to meet particular laboratory needs.

Zenie introduced two indices [24], the Economic Justification Index (EJI) and the Strategic Justification Index (SJI), to evaluate the effectivity of introduction of robots into a laboratory. EJI is the ratio of the annual hours saved and the average cost per hour versus the total installed cost. SJI is calculated from the following:

(1) *High-quality results* – Better precision than manual results, leading to higher quality products, more effective research and faster new product introductions.
(2) *Multiple application flexibility* – Ability to do multiple procedures, transfer proven procedures between laboratories, and the flexibility to reconfigure when needs change.
(3) *Faster turnaround of results* – More rapid availability of information leading to more timely decisions in research, product development, quality-control and process control.
(4) *Safety* – Isolation of people from hazardous environments or protection of critical experiments from human contamination.

Each of these factors is evaluated individually and the sum of the results gives us the combined justification for the project concerned. The use of these two indices is just an example of the justification procedure for introduction of robotics to a laboratory and each individual procedure can be set up with other factors to be evaluated and weighed.

Zenie also cited a survey of successful laboratory robotic installations conducted in 1988 [24]. Here are two out of several specific comments listed in the citation:

> 'Most important is picking the key person to implement the technology. That one person will make or break the success of the initial application'.

> 'The single most important factor in getting a system up and running and keep it running, in my experience, is to dedicate a person to the project'.

Both are emphasizing the importance of selection and assignment of a key person to fit the job.

As stated above, biosafety considerations should be taken into account when evaluating the introduction of robotics and this will become one of the most important factors when justification is made. In some cases, like biotechnological processes, we should be aware that contamination comes from operator contact to specimens or subjects, rather than *vice versa.*

### Impact of robotics employment

*Impact on the development of analytical systems: development of robotics friendly instrumentation*

In the past all laboratory analytical instruments have been developed and designed to be user friendly. Robotic systems replacing human tasks were developed to mimic human movements and were forced to adapt to existing instruments. Even if the instrument operation and sample entry is not convenient for robots, robots still have to be programmed to overcome these difficulties. Sometimes, it may even be necessary for a robot to enter commands on the keyboard of an instrument using its fingers just like a technician does.

However, if automation of a laboratory is to proceed to higher levels and wider areas of application, whether or not robots are actually employed, it is clear that some equipment will have to be developed to be 'friendly' towards the robotics of automation systems. For example, communication between robots, instruments and computers should be done on line. Sampling port configuration must be easily accessible by robots, and in some cases sample containers could be designed for easy handling by robots. However, these designs should not exclude human use operability, which will be needed in case of emergency or accidental shut down of the robotics system. At the Kochi Medical School some instruments were specially modified to meet the requirements to fit the belt-line system previously described.

A minimal communication interface hardware between instruments and robots and/or computer should be made available now. The products of different instrument and robot manufacturers should be compatible with each other and protocols should be standardized.

*Impact on clinical chemists and technologists and on economics*

The clinical laboratory is being exposed to increasing numbers of biohazardous specimens and in the future we will face higher risks in the clinical laboratory environment. It might be possible to employ technical staff who will work in such environments at higher rates of pay, but these personnel will become less available with time. We should consider the introduction of alternatives which will handle these risk environments rather better.

### Summary

Increasing numbers of robots are going to be employed in industrial chemical laboratories. Most of these will be used to reduce the monotonous tasks of sample preparation, to minimize human exposure to dangerous environments, or to carry out huge numbers of repetitive experimental procedures. For example, looking for the most effective condition or combination in chemical synthesis or the best micro-organism in a large number of cultures.

In the clinical laboratory the situation is slightly different and robotics is not so widely applied in clinical laboratories, but there is definite trend to employ robots or robotic systems both to reduce labour volume and exposure of employees to possible biohazards and to help get more precise and correct results. These needs will be hard to fulfil via the usual automated devices and especially when adequate devices are not available. Specially designed machines will have to be produced to satisfy these demands and robotics will play a part. Finally we need to evaluate the introduction of robotics in terms of economy, strategy, biosafety and other aspects.

Typical examples of implementation of robotics in the clinical laboratory are transportation of specimens, front-end automation of sample preparation separation and aliquotting as well as selected processes in a large scale automation system.

Robots that are currently commercially available are not intelligent enough to be easily handled by personnel who are not trained in robotics. So there is a need for people dedicated to robotics to join projects from the very beginning of the plan – this situation is likely to remain the same for some time.

A standarized interface and protocol for communication between robotics, computer and instrument should be established before robotics is widely employed.

### References

1. Felder, R. A., Boyd, J. and Savory, J., *Medical Laboratory Products* (July 1987), 18–19.
2. Zenie, F. H., *Advances in Laboratory Automation-Robotics* (Zymark Corporation, Hopkinton, Massachusetts, 1984).
3. Felder, R. A., Boyd, J. C., Savory, J., Margrey, K., Vaughn, D. and Martinez, A., *Proceedings of the IUPAC 3rd International Congress of Automation and New Technology in the Clinical Laboratory* (Blackwell Scientific Publications, 1990), 87–92.
4. Isenhour, T. L., Eckert, S. E. and Marshall, J. C., *Analytical Chemistry*, 61 (1989), 805A–814A.
5. Margrey, K. S., Martinez, A., Vaughan, D. and Felder, R. A., *Clinical Chemistry*, **36** (1990), 1572–1575.
6. MIDI 1.0 Detailed Specification Document Version 4.1. MIDI Manufacturers Association and Japan MIDI Standard Committee (1989).
7. Kramer, G. W., *Clinical Chemistry*, **36** (1990), 1556–1560.
8. Godfrey, O. W., Ford, L. M. and Eaton, T. E., *Program and Abstracts of Oak Ridge Conference on Advanced Analytical Concepts for the Clinical Laboratory 1990.* Paper No. 4.
9. De Moranville, V. and Ellis, J. E., *Clinical Chemistry*, **36** (1990) 1588–1590.
10. Seligson, D., *Clinical Chemistry*, **29** (1983), 1154.
11. Hawk, G. L., *Clinical Chemistry*, **30** (1984), 937.
12. Sasaki, M., *Rinshobyori*, **35** (1987), 1072–1078.
13. Sasaki, M. *Proceedings of the IUPAC 3rd International Congress on Automation and New Technology in the Clinical Laboratory* (Blackwell Scientific Publications, 1990), 97–101.
14. Sasaki, M., *Clinical Chemistry*, **35** (1989), 1052.
15. Sasaki, M. and Ogura, D. A., *Clinical Chemistry*, **36** (1990), 1567–1571.
16. Sasaki, M., *Rinshobyori*, **32** (1984), 119–126.
17. Felder, R. A., Margrey, K. S., Boyd, J. C. and Savory, J., *Clinical Chemistry*, **32** (1986), 1103.
18. Felder, R. A., Boyd, J. C., Martinez, A., Margrey, K. and Savory, J., *Clinical Chemistry*, **33** (1987), 960.
19. Felder, R. A., Barnwell, H. and Holman, W., *Clinical Chemistry*, **34** (1988), 1302.
20. Felder, R. A., Boyd, J. C., Savory, J., Margrey, K. S., Martinez, A. and Vaughn, D., *Clinics in Laboratory Medicine*, **8** (1988), 699–711.
21. Felder, R. A., *Clinical Chemistry*, **35** (1989), 1051.
22. Felder, R. A., Boyd, J. C., Margrey, K. S., Holman, W. and Savory, J., *Clinical Chemistry*, **36** (1990), 1534–1543.
23. Cross, J., *Advances in Laboratory Automation-Robotics* (Zymark Corporation, Hopkinton, Massachusetts, 1980), 321–337.
24. Zenie, F. H., *Advances in Laboratory Automation Robotics* (Zymark Corporation, Hopkinton, Massachusetts, 1988), 399.

